# Intractable hyponatremia secondary to syndrome of inappropriate antidiuresis complicated with empty sella: A case report

**DOI:** 10.1097/MD.0000000000033436

**Published:** 2022-04-07

**Authors:** Wenli Zheng, Shiqin Fan, Jie Chen, Jing Ma

**Affiliations:** a Department of Intensive Care Medicine, Liyuan Hospital, Tongji Medical College of Huazhong University of Science and Technology, Wuhan, Hubei, China.

**Keywords:** empty sella, hyponatremia, pulmonary infection, severe pneumonia, syndrome of inappropriate antidiuresis

## Abstract

Hyponatremia is a common electrolyte disorder in elderly critically ill patients, and it may be associated with poor outcomes, higher morbidity, and mortality. Syndrome of inappropriate antidiuresis (SIAD) is one of the main causes of hyponatremia, with an insidious onset that is highly misdiagnosed. Primary empty sella lesions are specific, mostly asymptomatic, and easily overlooked. SIAD combined with empty sella is much rarer in clinic, this article focuses on the diagnosis and management of an elderly patient with intractable hyponatremia secondary to syndrome of inappropriate antidiuresis complicated with empty sella.

**Patient concerns:**

An 85-year-old male patient with severe pneumonia presented with progressive and intractable hyponatremia.

**Diagnoses:**

The patient had clinical signs of persistent hyponatremia, low plasma osmolality, elevated urinary sodium excretion, and hyponatremia that worsened with increased intravenous rehydration and was effective with appropriate fluid restriction. The diagnosis of SIAD combined with empty sella was made in combination with the findings of the pituitary and its target gland function.

**Interventions:**

Numerous screenings were performed to clarify the cause of hyponatremia. His overall condition was poor due to recurrent episodes of hospital-acquired pneumonia. We treated with ventilation support, circulatory support, nutritional support, anti-infection, and continuous correction of electrolyte imbalance.

**Outcomes:**

His hyponatremia gradually improved through aggressive infection control, appropriate fluid restriction (intake controlled at 1500–2000mL/d), continuous electrolyte correction, supplementation with hypertonic salt solution, and potassium replacement therapy.

**Lessons:**

Electrolyte disorders, especially hyponatremia, are very common in critically ill patients, but the etiology of hyponatremia is challenging to diagnose and treat, and timely attention and proper diagnosis of SIAD and individualized treatment are the significance of this article.

## 1. Introduction

^[1]^ Hyponatremia has a high prevalence in acute and chronic hospitalized patients and may lead to increased length of stay and higher financial burden.^[2–8]^ It is a marker of disease severity and is associated with increased mortality.^[9]^ Syndrome of inappropriate antidiuresis (SIAD) is the most common cause of hyponatremia.^[10]^ SIAD is considered to be a group of clinical syndromes of low serum sodium, high urinary sodium, and high urinary osmolality caused by sustained release or enhanced activity of endogenous antidiuretic hormone (ADH) or mutations in the ADH receptor gene. SIAD has an insidious onset and is easily misdiagnosed. The cause of SIAD includes pulmonary disease (infection), malignancy, central nervous system disease, or various drugs.^[11]^ Empty sella (ES) is caused by subarachnoid herniation into the saddle and compression of the pituitary stalk,^[12]^ primary empty sella is mostly asymptomatic, but a few patients may have endocrine function abnormalities and compression symptoms such as headache and vision loss. SIAD combined with ES is much rarer in clinic, this article focuses on the diagnosis and management of an elderly patient with intractable hyponatremia secondary to syndrome of inappropriate antidiuresis complicated with empty sella, hoping to deepen the understanding and consideration of this type of disease by discussing this case.

## 2. Case report

An 85-year-old man was admitted to the rehabilitation department of our hospital 4 months ago with limited hip movement following “artificial hip replacement” for a left femoral head fracture and was transferred to the intensive care unit 1 month later with respiratory failure. He had a previous history of coronary artery disease; a 5-year history of chronic obstructive pulmonary disease without regular medication; and a history of tuberculous pleurisy 2 years ago, which resolved after 9 months of standardized antituberculosis treatment.

On admission his temperature was 36.5°C, heart rate was 138 beats per minute, respiration rate was 38 per minute, blood pressure was 51/32 mm Hg, and oxygen saturation was 81%; He was in a comatose state with cyanosis, bilateral pupils equal in size and roundness, blunted light reflex; respiratory sounds were coarse in both lungs and wheeze could be heard at full lung; cardiac arrhythmia, no pathological murmur was heard in each valve; the abdominal wall was soft, no abnormality was felt, and the auscultatory bowel sounds were weaker; muscle strength of extremities was uncooperative and muscle tone was reduced; the rest of the physical examination was within normal limits. Laboratory results: leukocytes 21.32*10^9/L, hemoglobin 111 g/L, serum sodium 134.7 mmol/L, albumin 35.1 g/L, ghrelin 6.4 U/L, ghrelin 17.6 U/L, creatinine 52.3 umol/L, uric acid 153.0 umol/L, glomerular filtration rate 92.1 minutes* 1.73 m^2, calcitoninogen 8.40 ng/mL, interleukin-6 111.69 pg/mL, ultrasensitive C-reactive protein 176.4 mg/L, brain natriuretic peptide 1273 pg/mL; blood gas analysis: PH 7.13, PCO2 94 mm Hg, PO2 89 mm Hg, lactate 1.6 mmol/L. He was diagnosed with respiratory failure, infectious shock, severe pneumonia, and hypoproteinemia, and was treated with tracheal intubation, mechanical ventilation, circulatory support, anti-infection, symptomatic, and nutritional support. Considering the patient’s advanced age and possible acute heart failure, his initial fluid intake was 2000 to 2500 mL and his urine output was 2183 to 3900 mL. He gradually developed hyponatremia, on the 7th day of admission his intake was 3000 mL and his serum sodium dropped to 130 mmol/L. Increasing the supplementation of isotonic salt solution worsened his hyponatremia, while the supplementation of concentrated sodium chloride solution did not significantly improve his serum sodium. His serum sodium fluctuated between 119 to 127 mmol/L during this period. Based on the formula, plasma osmolality = 2*(serum sodium + serum potassium) + blood glucose (both in mmol/L), his plasma osmolality was calculated as 260.73 mOsm/kg·H_2_O; urinary sodium was 380.89 mmol/24 hours (detailed data are shown in Table 1), so was his intractable hyponatremia considered as a diagnosis of SIAD or hypopituitarism or primary adrenocortical hypofunction? To find out the exact cause of the patient’s hyponatremia, we continued to refine relevant examinations. His tumor markers and rheumatic immune antibody tests showed no significant abnormalities, which initially ruled out malignancy. Bedside cardiac ultrasound showed: widened ascending aorta; mild aortic and mitral valve insufficiency; reduced left ventricular diastolic function; a small amount of pericardial cavity effusion; the ejection fraction was 56%. Bedside abdominal ultrasound showed multiple cysts in the liver; cysts in the right kidney. His bilateral adrenal computed tomography (CT) did not show obvious abnormality. His chest CT showed: post-tracheotomy, chronic bronchitis, and emphysema; infection of the lingual segment of the left upper lung, posterior segment of both upper lungs, and medial segment of the right middle lobe and both lower lungs; focal fibrosis of the lingual segment of the left upper lung, posterior segment of the right upper lung and medial segment of the right middle lobe; a small amount of pericardial effusion; bilateral pleural thickening and small bilateral pleural effusion. Genetic testing of his alveolar lavage fluid did not reveal mycobacterium tuberculosis. His cranial CT suggested bilateral frontal subcortical lacunar cerebral infarction. Intrasellar change, considering empty sella. Bilateral sphenoid sinusitis. Magnetic resonance imaging of the pituitary indicated: the pituitary was crescent-shaped and tightly close to the saddle floor, the pituitary height was 1.0 mm, and the pituitary stalk was displaced and inserted into the sellar floor. However, his target gland hormone levels were essentially normal, and there was no evidence of adrenal, thyroid, or aldosterone dysfunction and hepatic and renal insufficiency (as shown in Table 1), so hyponatremia due to hypopituitarism could be excluded. Combined with the above tests, the diagnosis of SIAD was consistent. As the disease progressed and the treatment plan was continuously adjusted, we observed that with the basic nutritional requirements, appropriate fluid restriction (FR) (meaning that the fluid intake was controlled at 1500–2000 mL/day and the FR had to be continued for some time before a significant therapeutic effect was seen) combined with hypertonic saline solution and aggressive potassium supplementation, his urine output was about 1000 to 1500 mL/day. His serum sodium gradually increased and could be maintained at 131 to 135 mmol/L.

**Table 1:** Biochemical and hormonal profile of our patient.

Biochemical	Values	Reference range
Serum sodium	124.6	137.0–147.0 mmol/L
Serum chlorine	90.7	99.0–110.0 mmol/L
Serum potassium	3.15	3.50–5.3 0 mmol/L
Glucose	4.47	3.90–6.12 mmol/L
Plasma osmolality	260.73	275–300 mOsm/Kg
Creatinine	33.5	57.0–111.0 umol/L
Blood urea nitrogen	4.47	3.60–9.50 umol/L
Uric acid	109.7	149.0–416.0 mmol/L
Blood phosphorus	0.57	0.85–1.51 umol/L
Urine specific gravity	1.025	1.005–1.030
Urine sodium	380.89	130–260 mmol/24 h
Urine potassium	52.11	25.00–100.00 mmol/24 h
Urine chlorine	393.24	170.00–250.00 mmol/24 h
ACTH	30.249	7.200–63.400 pg/mL
Cortisol	37.9 (7:00–9:00); 29.15 (15:00–17:00)	4.260–24.850 Ug/dL
Angiotensin II (prone)	167.224	25.000–129.000 pg/mL
Plasma renin (prone)	2.130	4.000–24.000 pg/mL
Aldosterone (prone)	88.409	10.000–160.000 pg/mL
TSH	1.76	0.35–5.10 mIU/L
FT3	2.69	11.20–23.80 Pmol/L
FT4	21.41	0.35–5.10 mIU/L

ACTH = adrenocorticotropic hormone, TSH = thyroid stimulating hormone, FT3 = free triiodothyronine, FT4 = free thyroxine.

## 3. Discussion

^[10]^ SIAD is considered to be a group of clinical syndromes of low serum sodium, high urinary sodium, and high urinary osmolality caused by sustained release or enhanced activity of endogenous ADH or mutations in the ADH receptor gene.^[13]^ It was first identified in 1957 by Schwartz WB in 2 patients with bronchopulmonary carcinoma. Currently, SIAD remains a diagnosis of exclusion, and the basic criteria required for diagnosis are as follows:^[14]^ decreased effective plasma osmolality (Posm<275 mOsm/kg·H_2_O); increased urinary osmolality (Uosm>100 mOsm/kg·H_2_O in hypotonicity); clinical euvolemia, as defined by the absence of signs of hypovolemia (orthostasis, tachycardia, decreased skin turgor, dry mucous membranes) or hypervolemia (subcutaneous edema, ascites); elevated urinary sodium excretion while on a normal salt and water intake (urinary sodium > 30 mmol/L); normal thyroid, adrenal, and renal functions; no recent diuretic use. SIAD needs to be differentiated from cerebral salt wasting syndrome (CSWS).^[15]^ CSWS is due to the increased release of natriuretic peptide, which increases renal sodium excretion along with increased water excretion. The diagnosis of CSWS requires evidence of inappropriate urinary salt loss and reduced effective arterial volume.^[16]^ Although both SIAD and CSWS are characterized by hyponatremia, low plasma osmolarity, high urinary sodium, and high urinary osmolality, differences in effective circulating blood volume and responsiveness to FR tests or rehydration tests are points of differentiation. SIAD requires FR, while CSWS requires adequate fluid and salt rehydration.^[17]^ Decreased blood urea nitrogen, creatinine, and uric acid levels are useful laboratory clues to diagnose volume failure; however, these indices may be influenced by other factors and are neither sensitive nor specific. High urine sodium and volume alone do not prove the presence of CSWS, as infusion of isotonic saline to patients with euvolemic SIAD also results in rapid excretion of sodium and fluid to maintain homeostasis.^[18]^ Gross P also noted the presence of mixed hyponatremia with hypovolemia complicated by siad. The patients suspected of having CSWS may also have coexisting SIAD. Sherlock M et al^[19]^ retrospectively analyzed 316 patients with subarachnoid hemorrhage and found that although SIAD was the primary cause of severe hyponatremia (69.2%), 4.8% of patients had severe hyponatremia due to the coexistence of CSWS and SIAD. Therefore, the patient’s volume status must be carefully assessed to differentiate between these disorders.

In this case, the patient was considered to have chronic severe hypotonic hyponatremia without diabetes mellitus, without the application of massive diuretics, after excluding cardiac, hepatic, and renal diseases, and after screening all medications that could cause hyponatremia. The patient’s plasma osmolality (<275 mOsm/kg·H_2_O), urinary sodium (<30 mmol/L), urinary osmolality (>100 mOsm/kg·H_2_O), low serum uric acid, creatinine, urea nitrogen, as well as appropriate FR resulting in improved serum sodium, are signs that support the diagnosis of SIAD. At this stage, SIAD and CSWS should be carefully identified. In patients with CSWS, the cornerstone of treatment is vigorous volume resuscitation and sodium replacement, but supplementation with isotonic saline solution negated CSWS, as the patient’s hyponatremia deteriorated significantly after intravenous infusion (serum sodium decreased from 126–120 mmol/L). So was ES the cause of his hyponatremia?

^[20]^ Empty sella syndrome (ESS) is a condition in which the pituitary gland is damaged after subarachnoid herniation into the sella turcica, which can be caused by increased intracranial pressure or dysfunction of the sellar diaphragm.^[11]^ Primary ESS is usually a radiological sign without any clinical significance found incidentally in patients with preserved pituitary function, while a few may present with endocrine or neurological disorders. In contrast, secondary ESS may have a history of tumor or radiotherapy, infection, trauma, and surgery in the pterygoid saddle area. There were no secondary factors in our patient, and primary ES was considered in combination with the pituitary magnetic resonance imaging findings and normal target gland hormone results.^[21]^ Although endocrine abnormalities are not the cause of hyponatremia in our patients, careful clinical evaluation of the neurological and endocrine manifestations associated with empty sella is required to differentiate it from SIAD. Alternatively, it may be explained that pituitary compression affects the level of ADH, but whether there is a causal relationship between empty sella and SIAD is lacking further evidence. In addition, although our patient’s adrenocorticotropic hormone was within the reference range, multiple cortisol measurements were elevated,^[22]^ which may be related to the presence and duration of stress. Cortisol levels can increase several-fold under stress.^[23]^ Cortisol is a physiological inhibitor of ADH secretion, which could explain the polyuria in our patient at the early stage of the disease.

^[4]^ Hyponatremia is multifactorial in most elderly hospitalized patients. The presence of SIAD combined with hyponatremia in this patient may be a combination of advanced age, prolonged bed rest, hypoalbuminemia, severe pneumonia, old tuberculosis, and the presence of vacuolated butterfly saddles. Aging is a risk factor for the occurrence of hyponatremia in elderly patients.^[24]^ Anpalahan M believes that the underlying mechanism of hyponatremia in patients with SIAD of unknown cause may be related to aging. Miller M et al^[25]^ and Shapiro DS et al^[4]^ in their respective studies have pointed out that even the relatively rare idiopathic SIAD is more common in the elderly. Elderly people have a reduced ability to regulate hyponatremia due to declining physical function and decreased sodium retention by the kidneys. The incidence of hyponatremia increases with age. Malnutrition is independently associated with hyponatremia. A study by Gómez-Hoyos E et al^[26]^ found severe malnutrition to be a risk factor for hyponatremia in critically ill patients. Although the mechanism is not clear in our case, the amount of low salt and fluid intake associated with undernutrition may exacerbate the negative balance of sodium.^[27–31]^ Pulmonary disease is also a common cause of SIAD, which can be associated with acute pneumonia, tuberculosis, chronic obstructive pulmonary disease, respiratory failure, and lung cancer.^[32]^ The mechanism of SIAD caused by pulmonary infection may be the action of an inflammatory cytokine (interleukin-6); hypoxemia and hypercapnia cause pulmonary vasoconstriction, increased resistance to the pulmonary circulation, reduced pulmonary blood volume, which acts on pressure receptors, resulting in right heart insufficiency, and reduced cardiac output, and reduced effective circulating blood volume; the disease continues to have a persistent effect on hypothalamic-pituitary function after pulmonary infection, and normal central regulatory mechanisms fail to precisely modulate the release of arginine vasopressin (AVP). If the cause of SIAD remains undetectable, the possibility of a tumor cannot be completely excluded.^[30]^ Martinez-Maldonado reported a 52-year-old woman diagnosed with idiopathic SIAD who was found to have pulmonary malignancy after up to 7 months. Gudsoorkar PS et al^[33]^ reported a case in which it took 2.5 years of extensive investigations before the cause of SIAD was found to be a neurocytoma of the maxillary sinus. A 9-year retrospective cohort study by Chih-Yang Hsu et al^[34]^ found that patients with SIAD of unknown causes accounted for 39.2%. Even severe hyponatremia will be well tolerated if the patient’s serum sodium decreases gradually over a period. Therefore, the presence of an underlying pathophysiological process should be considered in patients with SIAD whose etiology cannot be clarified, and follow-up needs to be continued for at least 1 year. Thus, it is speculated that pulmonary infection may be a predisposing factor for his SIAD coexisting with hyponatremia. Multi-drug-resistant bacteria were cultured several times during the course of his illness, and although the infection was intermittently controlled, his hyponatremia persisted. The mechanism of interaction between pulmonary infection and SIAD is inconclusive and still needs to be explored in depth in the future.

There is individual variation in the effectiveness of SIAD treatment due to patient intolerance and the fact that most are not etiologically specific.^[17]^ Treatment options for SIAD include FR, supplementation with hypertonic salt solutions, AVP receptor antagonists, tab diuretics, urea, demeclocycline, and lithium.^[17,35]^ Although FR is the first-line treatment for hyponatremia in SIAD, its effectiveness remains highly controversial. An observational study by Verbalis JG et al^[36]^ of 1524 patients with SIAD from the hyponatremia registry found that FR was ineffective in more than half of cases.^[37]^ A similar study also showed the limited benefit of FR in the routine treatment of SIAD, with plasma sodium elevated by only 2 mmol/L on the first day, which was not statistically different compared with no treatment. Verbalis JG et al^[17]^ also recommended adjusting the threshold for serum sodium to < 131 mmol/L, mainly to avoid raising serum sodium by restricting fluids. We all know that volume failure caused by various reasons is unhelpful.^[17]^ The American consensus considers high urinary osmolality (>100 mOsm/kg·H_2_O), the sum of urinary sodium and urinary potassium concentrations over serum sodium concentration as predictors of increased likelihood of FR failure in patients with SIAD, and the situation in our patient is compatible with both conditions. Due to the tracheotomy his daily occult water loss from the airway was about 1000 mL and his sputum was large and thick, requiring airway humidification to thin the sputum. He did not appear to have clinical signs of volume overload and even signs of volume contraction (bedside ultrasound suggested that his inferior vena cava was relatively narrow). During the course of the disease, he had hypoalbuminemia (35.1–24.6 g/L) and to ensure basic caloric needs, we believe that he could not tolerate strict FR. Cuesta M et al^[38]^ also concluded that for the correction of serum sodium in SIAD patients with pneumonia, it is wise to maintain proper blood pressure and blood volume by rehydration as opposed to FR. Therefore, the optimal fluid therapy for SIAD associated with pneumonia should continue to be explored in prospective studies in the future. Combined medication may be used if the FR is not effective.^[39]^ Arginine pressor AVP receptor antagonists promote water excretion without increasing urinary sodium loss by competitively binding to the AVP V2 receptor on the renal collecting duct. However, the effective therapeutic dose of this drug varies greatly among individuals, so the patient’s serum sodium should be closely monitored and the dose should be adjusted promptly.^[40]^ Because too rapid a correction of hyponatremia may cause central nerve demyelination and aggravate brain cell damage. Sood L et al^[41]^ even proposed the use of hypertonic saline in conjunction with desmopressin as a protocol to predict and control the rate of sodium correction.^[42,43]^ In hyponatremic patients with combined craniosynostosis where salt and fluid therapy is difficult, salt corticosteroids (fludrocortisone) may be recommended to increase renal tubular sodium reabsorption; however, we are unavailable because it is not yet approved for marketing in China. Shen et al^[44]^ reported 4 patients with refractory hyponatremia complicated with SIAD and CSWS after TBI, and a triple regimen of intravenous rehydration, hydrocortisone, and tachyphylaxis resulted in normalization of serum sodium. Our patient was also treated with intravenous hydrocortisone for 3 days, his hyponatremia did improve significantly, but we did not continue the hormone given the patient’s multiple cultures of multi-drug resistant bacteria and his previous history of tuberculosis. After discontinuation of the hormone, his serum sodium was again not promising.^[45]^ No single therapy can represent the optimal treatment for all patients.

## 4. Conclusion

The choice of treatment strategy for hyponatremia in SIAD patients should be individualized.

Hyponatremia is a common type of clinical electrolyte disorder, and most studies have shown that chronic hyponatremia may impair local neurological function and increase the risk of death. The presence of hyponatremia should be taken seriously in clinical work, for patients diagnosed with SIAD combined with hyponatremia, a comprehensive screening of possible etiologies should be conducted, and potential causes should be closely followed up to achieve early detection and early individualized treatment.

## Acknowledgments

The study was approved by the Ethics Committee of Liyuan Hospital, Affiliated to Tongji Medical College, Huazhong University of Science and Technology, Wuhan, China; informed consent was obtained from the patient’s family.

## Author contributions

**Data curation:** Wenli Zheng, Shiqin Fan, Jie Chen.

**Formal analysis:** Wenli Zheng.

**Investigation:** Wenli Zheng, Shiqin Fan.

**Methodology:** Wenli Zheng.

**Writing – original draft:** Wenli Zheng, Shiqin Fan.

**Writing – review & editing:** Wenli Zheng, Jing Ma, Shiqin Fan.
